# Energetics and Electronic Structure of Triangular Hexagonal Boron Nitride Nanoflakes

**DOI:** 10.1038/s41598-018-34874-x

**Published:** 2018-11-09

**Authors:** Mina Maruyama, Susumu Okada

**Affiliations:** University of Tsukuba, Graduate School of Pure and Applied Sciences, Tsukuba, 305-8571 Japan

## Abstract

We studied the energetics and electronic structures of hexagonal boron nitrogen (h-BN) nanoflakes with hydrogenated edges and triangular shapes with respect to the edge atom species. Our calculations clarified that the hydrogenated h-BN nanoflakes with a triangular shape prefer the N edges rather than B edges irrespective of the flake size. The electronic structure of hydrogenated h-BN nanoflakes depends on the edge atom species and their flake size. The energy gap between the lowest unoccupied (LU) and the highest occupied (HO) states of the nanoflakes with N edges is narrower than that of the nanoflakes with B edges and the band gap of h-BN. The nanoflakes possess peculiar non-bonding states around their HO and LU states for the N and B edges, respectively, which cause spin polarization under hole or electron doping, depending on the edge atom species.

## Introduction

Hexagonal boron nitride (h-BN) is known to be a prototypical layered material in which each layer is composed of B and N atoms alternately arranged in an hexagonal network similar to that of graphite^1–4^. Along the direction normal to the layer, in sharp contrast to graphite, each layer is weakly bound in an AA’ arrangement, in which N atoms are situated just above/below B atoms in adjacent layers, and vice versa, owing to the interlayer Coulomb interaction between B and N atoms. According to the layered structure, an individual atomic layer of h-BN can be synthesized as in the case of other layered materials, such as graphite^5,6^ and transition metal chalcogenide compounds^7,8^. The chemical difference between B and N atoms make h-BN an insulator with a large energy gap of approximately 5 eV between the top of the valence band and the bottom of the conduction band, localized on N and B atoms, respectively^9–12^. Analogous with graphene, a h-BN sheet can form various derivatives with different shapes and dimensions by imposing appropriate boundary conditions. Nanoscale tubes have been synthesized and their physical properties have been investigated. These studies showed that the insulating electronic properties and band gap of such nanotubes are insensitive to their chirality and diameter^13–16^, which is in sharp contrast to carbon nanotubes^17,18^. h-BN can also form polycyclic structures with nanometer sizes, as can carbon^19,20^. In addition to small molecules, such as borazine and small polycyclic borazine derivatives, h-BN with a triangular shape and a size of several nanometers has been synthesized on appropriate substrates by chemical vapor deposition (CVD)^21–23^. Scanning transmission electron microscopy experiments have clarified that the triangular nanoflakes possess zigzag edges of N atoms, even though the edge formation energy of hydrogenated h-BN nanoflakes is insensitive to their edge angle and edge atom species^24^.

From a topological view, polycyclic materials consisting of hexagonal networks have been attracting much attention owing to their electronic structures for two decades. Graphene nanoribbons with hydrogenated zigzag edges possess peculiar edge localized states at the Fermi level and in the one-dimensional Brillouin zone, because of the delicate balance of electron transfer among the atomic sites near the edges^25–29^. In addition to the edge structure, the shapes of the C nanoflakes causes further variation in their electronic structures^30^. The sp^2^ C nanoflakes with triangular shapes and zigzag edges have non-bonding states at the Fermi level leading to the high spin ground state, in which the number of unpaired electrons corresponds to the number difference between two sublattices: phenalenyl, consisting of three benzene rings (C_13_H_9_), possesses a *S* = 1/2 ground state and triangulene, consisting of six benzene rings (C_22_H_12_), has a *S* = 1 triplet ground state^31–39^. In addition to the isolated molecules, the triangular sp^2^ C domain embedded into h-BN also exhibits a similar spin polarized state as their ground state, which slightly depends on the border atom species^40^. Because of the structural similarity of h-BN to graphene, h-BN nanoflakes with triangular shapes may also possess a similar non-bonding state near their occupied state and unoccupied state edges, depending on the edge terminations^21^. However, the detailed electronic structure of such h-BN nanoflakes with triangular shapes is still unclear with respect to the edge terminations.

In this work, we aim to investigate the energetics and electronic structure of triangular h-BN nanoflakes with respect to their sizes and edge terminations to provide theoretical insight into the formation mechanisms of triangular h-BN with hydrogenated N edges during CVD experiments. Furthermore, we also explore the possibility of spin polarization of triangular h-BN nanoflakes associated with the saturated non-bonding states by injecting carriers under an external electric field. Our calculations showed that triangular h-BN nanoflakes with hydrogenated N edges are more stable than those with hydrogenated B edges for all the flake sizes studied here and for any B sources. The energy gap between the highest occupied (HO) and the lowest unoccupied (LU) states of the flakes with hydrogenated N edges are narrower than not only those with hydrogenated B edges but also the band gap of the two-dimensional (2D) h-BN, because the LU state possesses a nearly free electron state^41–44^ distributed outside and alongside the edge atomic sites. The electron states of the nanoflakes with N edges around the HO states possess a non-bonding nature for the flakes with hydrogenated N edges, which causes spin polarized ground states under hole doping by the gate electrode.

## Results

**Figure 1:**
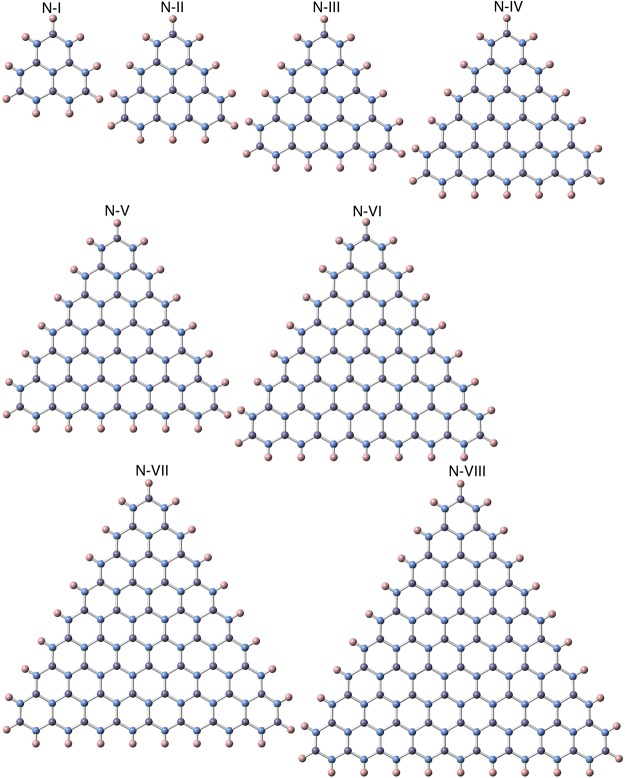
Optimized structures of triangular h-BN nanoflakes with an hydrogenated N edge. Violet, blue, and pink balls represent B, N, and H atoms, respectively.

**Figure 2:**
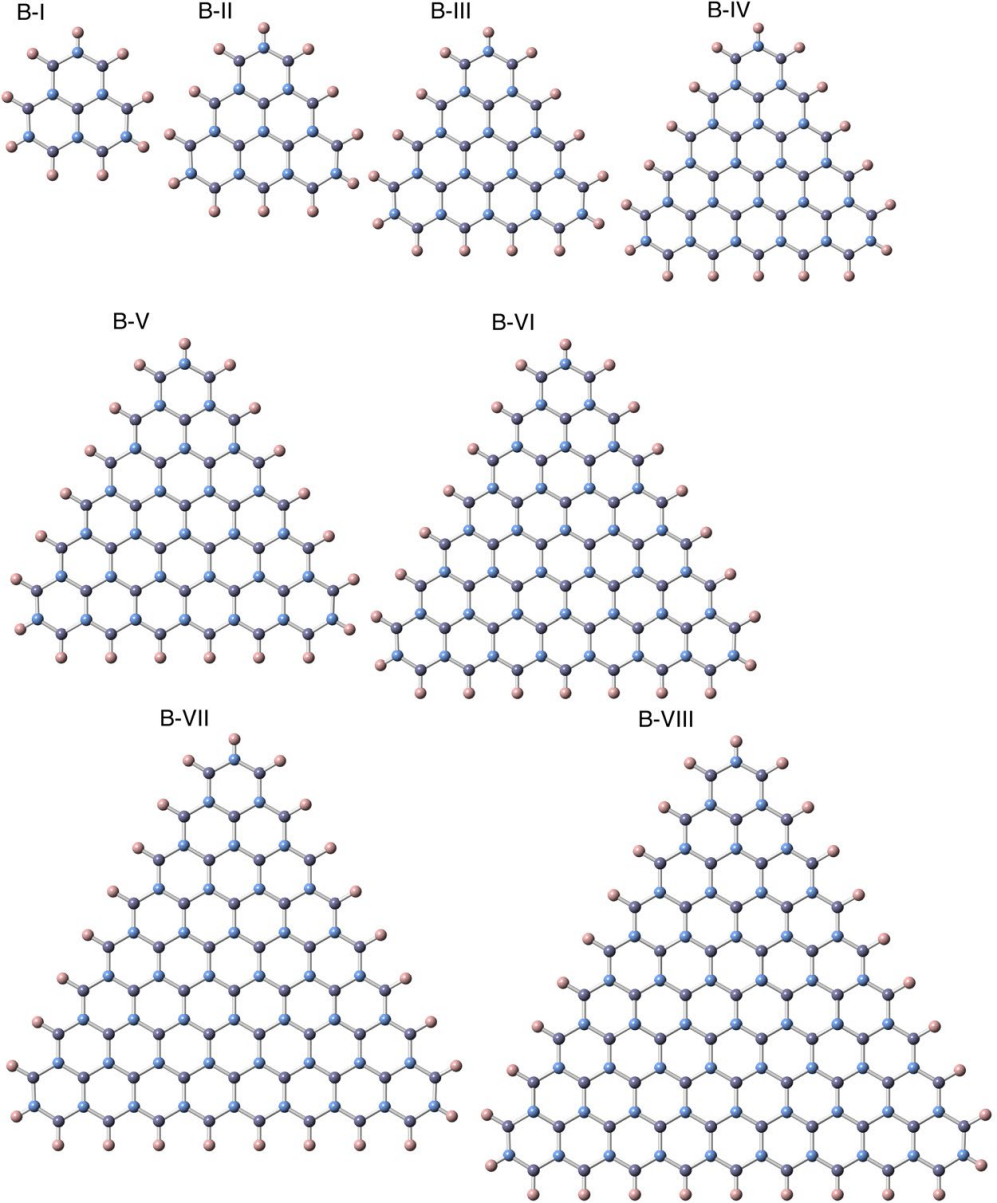
Optimized structures of triangular h-BN nanoflakes with an hydrogenated B edge. Violet, blue, and pink balls represent B, N, and H atoms, respectively.

Figures 1 and 2 show the optimized structure of triangular h-BN nanoflakes with hydrogenated N and hydrogenated B edges, respectively, consisting of 3∼45 hexagonal BN rings. The flakes with both N and B edges were found to retain their triangular shape after structural optimization, irrespective of their sizes and edge terminations. Furthermore, each BN ring in the nanoflakes also kept its hexagonal shape for both the inner and edge regions. The detailed bond lengths of the triangular nanoflakes with hydrogenated N and B edges are summarized in Table 1. Bond alternation induced by the edges occurred in the nanoflakes, even though the BN bonds located at three corners of the flakes were slightly shorter than the other BN bonds. The optimum bond length of corner bonds was 0.143 nm for all edge lengths. In contrast, the BN bond at the edges and inner region of the nanoflakes was 0.144∼0.146 nm, which was close to the bond length of h-BN.

**Table 1:** Optimized edge lengths and bond length [nm] of the triangular h-BN nanoflake with hydrogenated N and B edges.

Structure	Edge length	BH	NH	BN (corner)	BN (edges)	BN (inner molecular bond)
N-I	0.713	0.120	0.102	0.143	0.144	0.146
N-II	0.965	0.120–0.121	0.102	0.143	0.144–0.145	0.145–0.146
N-III	1.216	0.120	0.102	0.143	0.144–0.145	0.145–0.146
N-IV	1.468	0.120	0.102	0.143	0.144–0.145	0.145–0.146
N-V	1.719	0.120–0.121	0.102	0.143	0.144–0.145	0.145–0.146
N-VI	1.972	0.120–0.121	0.102	0.143	0.144–0.145	0.145–0.146
N-VII	2.222	0.120	0.102	0.143	0.144–0.145	0.145–0.146
N-VIII	2.474	0.120	0.102	0.143	0.144–0.145	0.145–0.146
B-I	0.671	0.120	0.102	0.143	0.145	0.146
B-II	0.921	0.120	0.102	0.143	0.145	0.145–0.146
B-III	1.173	0.120	0.102	0.143	0.145	0.145–0.146
B-IV	1.424	0.120	0.102	0.143	0.145	0.145–0.146
B-V	1.675	0.120	0.102	0.143	0.145	0.145–0.146
B-VI	1.925	0.120	0.102	0.143	0.145	0.145–0.146
B-VII	2.176	0.120	0.102	0.143	0.145	0.145–0.146
B-VIII	2.427	0.120	0.102	0.143	0.145	0.145–0.146
2D h-BN	—	—	—	—	—	0.144

**Figure 3:**
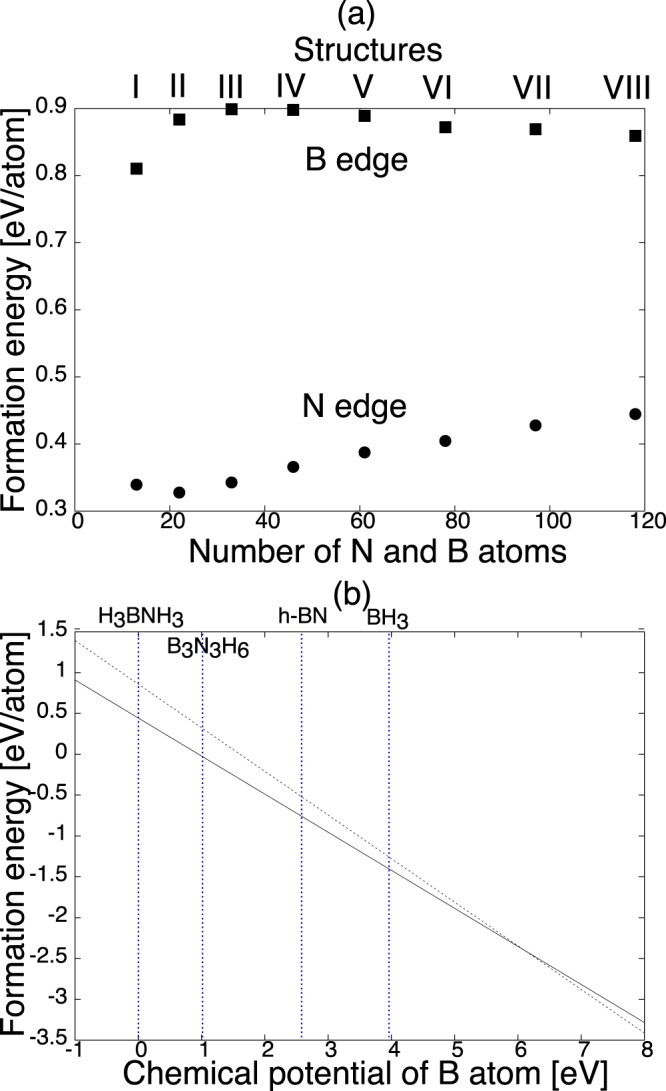
(**a**) Formation energy per atom of triangular h-BN nanoflakes with hydrogenated B or N edges as a function of the nanoflake sizes corresponding to the number of B and N atoms. Circles and squares denote the energy of the nanoflakes with N and B edges, respectively. (**b**) Formation energy per atom of the triangular h-BN nanoflakes of N-VIII and B-VIII as a function of the chemical potential of the B atom. Solid and dotted lines denote the energy corresponding to N-VIII and B-VIII, respectively. Vertical lines indicate the chemical potential of various molecules containing B atoms.

Figure 3(a) shows the calculated formation energy per atom of the triangular h-BN flakes with hydrogenated N and B edges as a function of the number of B and N atoms. The formation energy, *ε*, is defined as

$$\varepsilon =\frac{{E}_{total}-{N}_{B}{\mu }_{B}-{N}_{N}{\mu }_{N}-{N}_{H}{\mu }_{H}}{{N}_{B}+{N}_{N}}$$

where *E*_*total*_ is the total energy of the triangular nanoflakes, *N*_N_, *N*_B_, and *N*_H_ are the numbers of N, B, and H atoms, respectively, and *μ*_N_, *μ*_B_, and *μ*_H_ are the chemical potentials of the N, B, and H atoms, respectively. The chemical potentials of B, N, and H were calculated by the total energies of ammonia borane (H_3_BNH_3_), N_2_ molecule, and H_2_ molecule, respectively. Note that the chemical potential of H is not a tunable parameter for investing the formation energy of the nanoflakes, because all edge atomic sites must be perfectly terminated by H atoms. The formation energy of the nanoflakes with hydrogenated N edges were lower by approximately 0.4 eV/atom than those with the hydrogenated B edges for all nanoflakes studied here. Thus, the preferential formation of the N edge of the triangular BN nanoflakes was ascribed to their energetic stability. Note that the formation energy of the nanoflakes with B edges was insensitive to their size. The energy retained a constant value of approximately 0.8 eV/atom except the energy of the smallest flake (B-I). For the N edges, the energy slightly increased with increasing the flake size and seemed to saturate at the energy of 0.45 eV/atom for the flakes with an edge length of 2.5 nm or longer. This indicated that the size selectively was absent for the formation of h-BN nanoflakes under the hydrogen-rich conditions.

It is worth investigating how the formation energy depends on the chemical potential of source molecules. Figure 3(b) shows the formation energy of the largest triangular h-BN flakes studied here with hydrogenated N and B edges (N-VIII and B-VIII) as a function of the chemical potential of the B atom. The chemical potential of the B atom was 0.00, 1.01, 2.60, and 4.00 eV for ammonia borane, borazine (B_3_N_3_H_6_), h-BN, and borane (BH_3_), respectively. For all chemical potentials of the B sources, the nanoflakes with hydrogenated N edges were more stable than those with hydrogenated B edges. This implied that less stable B sources are necessary for the selective synthesis of the triangular BN flakes with B edges.

**Figure 4:**
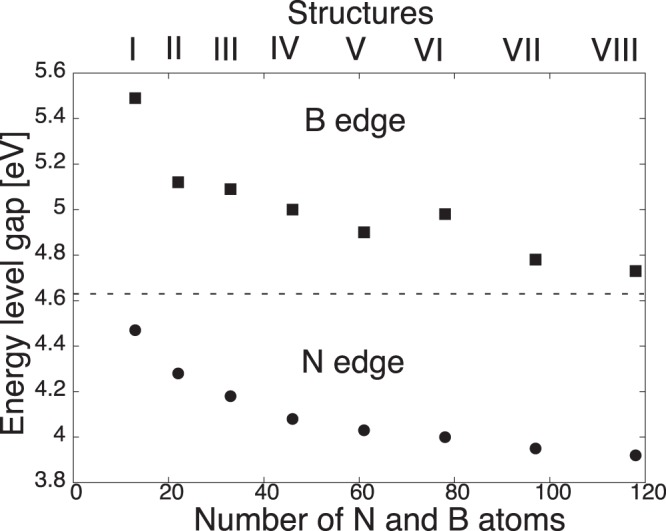
Energy gap between HO and LU states of the triangular h-BN nanoflakes as a function of the nanoflake size or the number of B and N atoms. Circle and squares denote the gap of the nanoflakes with hydrogenated N and B edges. The horizontal dotted line denotes the band gap of an isolated 2D h-BN sheet calculated by DFT with GGA.

Figure 4 shows the energy gap between the HO and LU states of the triangular h-BN nanoflakes as a function of their sizes. All nanoflakes had a large gap of approximately 4~5 eV between the HO and LU states. The gap was sensitive to the flake size and edge termination. The gap of the nanoflakes with N edges was narrower than that of the nanoflakes with B edges and the band gap of the 2D h-BN. Furthermore, the gap gradually decreased with increasing the number of atoms and saturated at approximately 3.92 eV for the flakes of N-VII or larger. In contrast, the gap of the nanoflakes with B edges monotonically decreased and asymptotically approached the band gap value of 2D h-BN with increasing the flake size. The anomalous gap profile of the triangular nanoflakes with hydrogenated N edges implied that the nanoflakes possessed an unusual electronic structure around the HO and LU states, which was completely different from those of h-BN and the nanoflakes with hydrogenated B edges.

**Figure 5:**
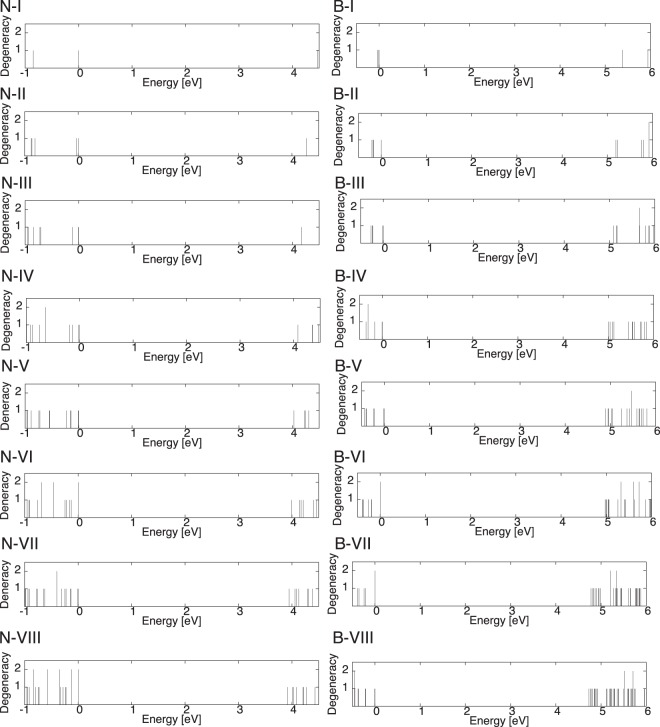
Electronic energy level of the triangular h-BN nanoflakes with hydrogenated N or B edges. The energy is measured from the highest occupied state.

Figure 5 shows the electronic structure of the triangular h-BN nanoflakes with hydrogenated N and B edges. For the nanoflakes with N edges, the number of states near the HO state increased with increasing the size of the nanoflake. Furthermore, the number of states corresponded to the difference between the numbers of N and B atoms: The non-degenerated LU state emerged for the nanoflake N-I. It has been clearly seen that two, three, four, and five states bunch up in the valence band edges including HO states for the nanoflakes N-II, N-III, N-IV, and N-V, respectively. For the nanoflakes with longer edges (the nanoflakes of N-VI, N-VII, and N-VIII), the number of bunching states was larger than that of the difference between the numbers of N and B atoms, because of the increase of the electron states near the gap. The LU state possessed a non-degenerate and isolated nature for all the nanoflakes with N edges.

In contrast to the nanoflakes with N edges, the electronic structure of the triangular nanoflakes with hydrogenated B edges exhibited an opposite nature: The electron states at and near the LU state exhibited a bunching nature while the states at and near the HO states were insensitive to the flake size. In the case of the valence states in the flakes with N edges, the states at and near the LU state bunched up, which corresponded to the difference between the numbers of B and N atoms. These facts implied that these bunched states in the valence and conduction state edges for the nanoflakes with N or B edges were associated with the non-bonding states of hydrocarbon molecules with triangular shapes.

**Figure 6:**
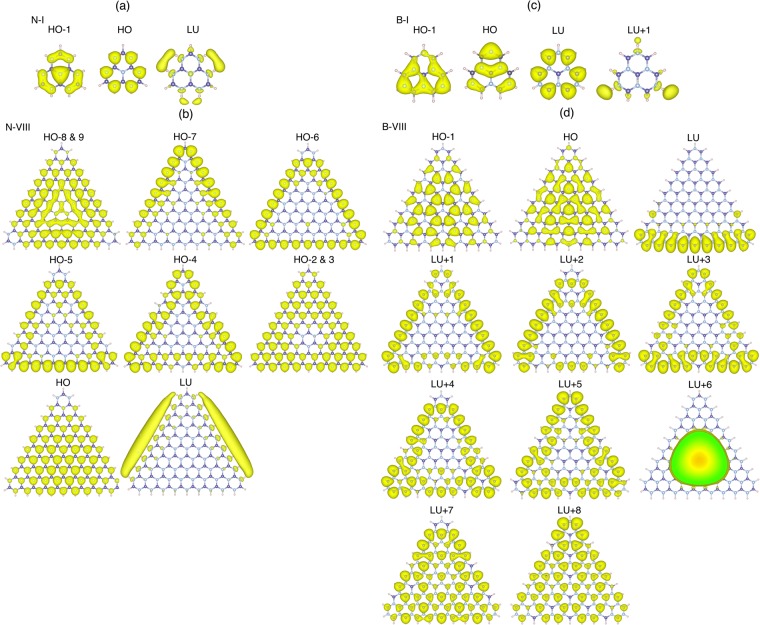
Isosurfaces of the squared wave function of the electronic states near the valence and conduction states edges of the triangular h-BN nanoflakes with (**a**) and (**b**) hydrogenated N edges (N-I and N-VIII) or (**c**) and (**d**) B edges (B-I and B-VIII).

To clarify the physical mechanism of the anomalous band gap profile of the nanoflakes with N edges as well as the bunched states near the band edges, we investigated the squared wavefunction near the band edges of the smallest and the largest triangular nanoflakes with N (N-I and N-VIII in Fig. 6(a) and (b)) and B (B-I and B-VIII in Fig. 6(c) and (d)) edges. For the smallest nanoflakes with N edges, the HO state exhibited a non-bonding nature where the wavefunction was distributed on the edge N atoms. In contrast, the HO − 1 state exhibited a bonding nature that extended throughout the flake. For the largest nanoflakes with N edges, the HO and near the HO states also exhibited a non-bonding nature. The HO state was a doubly degenerate state which was distributed on N atoms throughout the flakes with a non-bonding nature. The states gradually increased their localized nature on the edge N atoms with a decrease of their eigenvalue: The distribution of the non-bonding state of the HO − 2 and HO − 3 states were slightly dislodged to the edge atomic sites, and the deeper occupied states (HO − 6 and HO − 7) were perfectly localized at the edge N atom, as is observed for the edge state of a graphene ribbon with zigzag edges. As for the LU state of the nanoflakes with N edges, the state exhibited a peculiar nature: The state was distributed outside and alongside the edge atomic site as in the cases of the nearly free electron state of graphene and h-BN^41–44^. A strong dipole of the N-H bond may cause a substantial downward shift of this unusual state which would lead to a narrow energy gap between the HO and LU states compared with the band gap of h-BN.

However, for the nanoflakes with B edges, the LU and lower unoccupied states exhibited a non-bonding nature, which were distributed on the B atom: The LU state was completely localized at the edge B atomic site, while the states gradually penetrated the inner B atomic site with an increase of their eigenvalues. The state 1 eV above the LU state was distributed on all B atomic sites with a non-bonding nature. The HO state exhibited a different nature to that of the nanoflakes with an N edge. The HO state of the nanoflakes with B edges was primarily localized on an N atomic site with a bonding nature. Therefore, in terms of the electron state around the gap, the electronic states of the triangular nanoflakes with hydrogenated B edges exhibited similar characteristics as those of h-BN except for unoccupied non-bonding states, which led to the fact that the HO-LU gap of the nanoflakes asymptotically approached the band gap of h-BN.

The non-bonding nature of the electron state near the valence band edge of the triangular h-BN nanoflakes with N edges implied that the flakes may possess spin polarized states as the ground state upon hole injection, as is the case of the half-filled state of polycyclic hydrocarbon molecules with a triangular shape. Table 2 summarized the number of unpaired electrons Δ*ρ* ( = *ρ*_*α*_ − *ρ*_*β*_, where *ρ*_*α*_ and *ρ*_*β*_ are the electron density of the *α* and *β* spins) of the triangular h-BN nanoflakes with hydrogenated N edges, N-I, N-II, N-III, and N-IV, under the hole concentrations from partial to full doping of the occupied states with a non-bonding nature. The number of unpaired electrons is proportional to the number of holes injected into the non-bonding states up to half-filling: The ground states of the nanoflakes N-I, N-II, N-III, and N-IV were *S* = 1/2, 1, 3/2, and 2, respectively, under the hole concentration corresponding to the half-filling of the non-bonding states near the valence band edge. Furthermore, the polarized spin gradually decreased with the further increase of the hole and vanished when the electron was fully removed from the non-bonding states.

**Table 2:** Dependence of the number of the polarized electron spin (Δ*ρ*) of N-I, N-II, N-III, and N-IV on the number of holes. Number in parentheses denotes the number of states with a non-bonding nature.

Number of holes	Δ*ρ*			
	I (1)	II (2)	III (3)	IV (4)
1	1	1	1	1
2	0	2	2	2
3	—	1	3	3
4	—	0	2	4
5	—	—	0	3
6	—	—	0	2
7	—	—	—	1
8	—	—	—	0

**Figure 7:**
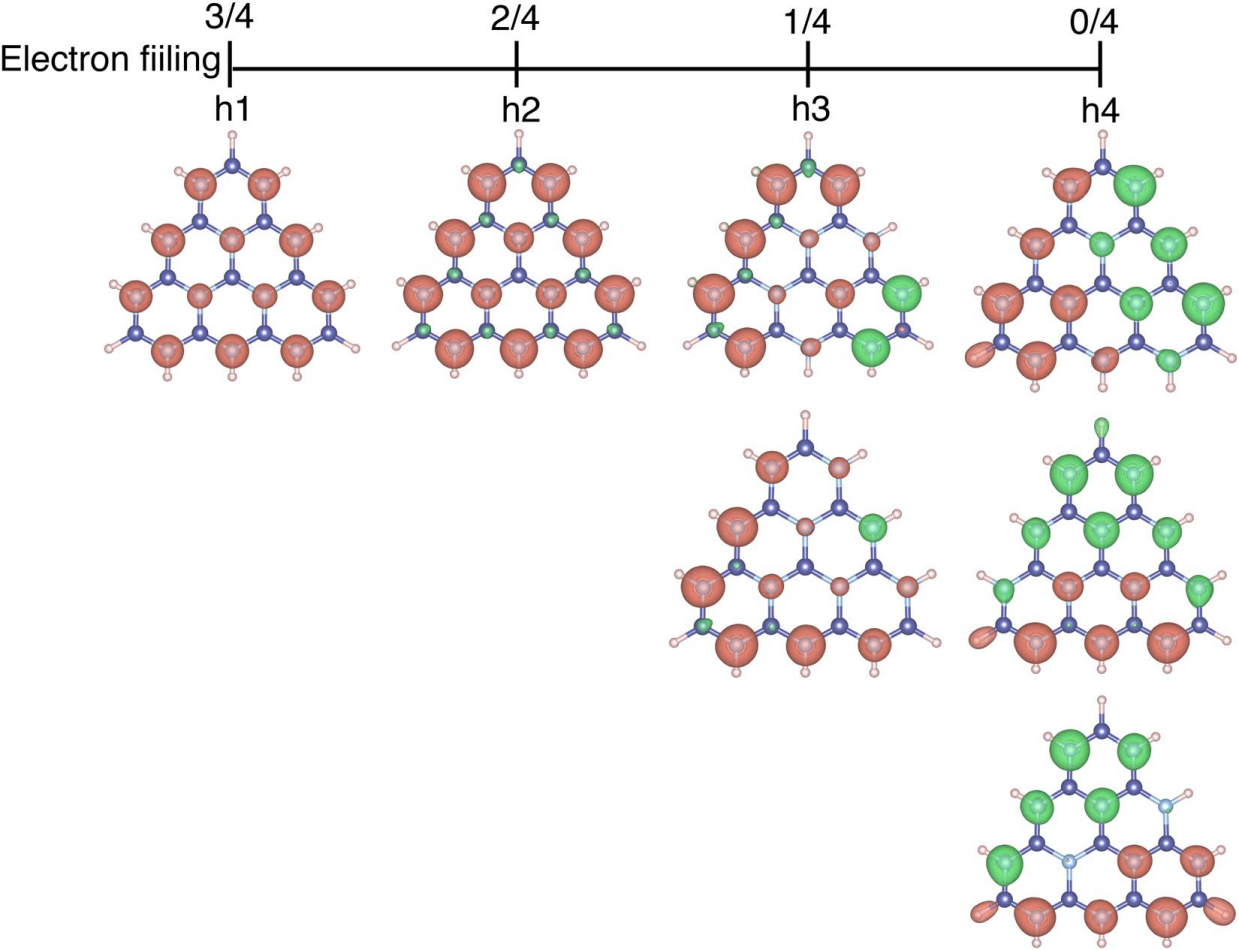
Isosurfaces of the spin density of the N-II nanoflake. Red and green isosurfaces indicate the *α* and *β* spin components.

Figure 7 shows the isosurfaces of the spin density ${\rm{\Delta }}\rho (\overrightarrow{r})$ of a triangular h-BN nanoflake (N-II) under hole concentrations of 1 h, 2 h, 3 h, and 4 h. For all hole concentrations, the nanoflakes possessed magnetic spin ordering which depended on the hole concentration. The polarized electron spins were ferromagnetically aligned throughout the N atomic sites up to the hole concentration of 2 h, which corresponded to the half filling of the non-bonding states near the HO states. The spin distribution was equivalent to the wavefunction distribution of the non-bonding states of the nanoflakes. In contrast, under the high hole concentrations, the N-II nanoflake possessed two and three spin polarized states for 3 h and 4 h doping, respectively. In both cases, the polarized electron spin had a multi domain structure in which the polarized spins were ferromagnetically distributed in each domain and antiferromagnetically aligned between spin domains. Despite the spatial distribution of spin states being different to each other, the total energies of these spin states were degenerate. Thus, various spin polarized states emerged as the metastable states of the triangular h-BN nanoflakes with N edges under a high hole concentration.

## Conclusions

In this work, we investigated the geometric and electronic properties of triangular h-BN nanoflakes with hydrogenated N or B edges using density functional theory with the generalized gradient approximation, to provide theoretical insight into the preferential formation of triangular flakes with N edges in CVD experiments. Our calculations showed that triangular h-BN nanoflakes with hydrogenated N edges are more stable by approximately 0.5 eV per atom than those with hydrogenated B edges for all flake sizes studied here and for any B source. The preferential synthesis of the nanoflakes with N edges in the CVD experiment is ascribed to their energetic stability. The electronic structure of the triangular h-BN nanoflakes strongly depends on the edge termination. The energy gap between the highest occupied (HO) and the lowest unoccupied (LU) states of the flakes with hydrogenated N edges are narrower than not only those with hydrogenated B edges but also the band gap of the 2D h-BN sheet, because the LU state possesses a nearly free electron state distributed outside and alongside the edge atomic sites. In contrast, the HO-LU gap of the nanoflakes with B edges asymptotically approaches the band gap of the 2D h-BN sheet with an increase of their flake size. Detailed electronic structure analysis near the occupied and unoccupied state edges clarifies that the triangular nanoflakes possesses a number of non-bonding states corresponding to the number difference between B and N atoms. For the nanoflakes with N edges, the non-bonding state emerges in the HO and just below it with a fully occupied nature. In contrast, for the nanoflakes with B edges, the non-bonding state emerges in the LU state and above it with an unoccupied nature.

According to the fully occupied non-bonding states, the triangular h-BN nanoflakes may exhibit spin polarization upon carrier injection into these states. To explore this possibility, we investigated the magnetic properties of the nanoflakes with hydrogenated N edges in terms of the hole doping in the FET structure using the ESM method. Under the hole injection, the nanoflakes exhibit spin polarized states as their ground state, where the number of unpaired electrons is proportional to the number of holes injected into non-bonding states up to a half-filling. The largest spin moment of the nanoflakes is *S* = *n*/2, where *n* is the number of nonbonding states, under the hole concentration corresponding to a half-filling of the non-bonding states. We also found a peculiar spin polarized state in the nanoflakes under a high hole concentration.

## Methods

All calculations were based on density functional theory^45,46^ as implemented in the program package Simulation Tool for Atom TEchnology (STATE)^47^. We used the generalized gradient approximation with the Perdew-Burke-Ernzerhof functional^48^ to describe the exchange-correlation potential energy among interacting electrons. Ultrasoft pseudopotentials generated by the Vanderbilt scheme were adopted as the interaction between electrons and nuclei^49^. Valence wavefunctions and the deficit charge density were expanded in terms of plane wave basis sets with cutoff energies of 25 and 225 Ry, respectively, which provided a sufficient convergence in the total energy and electronic structure of the h-BN related nanostructures. Brillouin zone integration was performed using Γ point sampling. The geometric structures of monolayer h-BN nanoflakes were fully optimized until the force acting on each atom was less than 1.33 × 10^−3^ HR/au. To simulate an isolated triangular h-BN nanoflake, each nanoflake was separated from its adjacent periodic images by at least 0.49 or larger and 0.70 nm for the lateral and normal directions.

We considered triangular h-BN nanoflakes (Figs 1 and 2) with edge lengths from 0.71 to 2.47 nm for hydrogenated N edges and from 0.67 to 2.43 nm for hydrogenated B edges (Table 1), which corresponded to the flakes containing 3~45 hexagonal rings. This enabled a quantitative analysis of the energetics and electronic structures of triangular h-BN nanoflakes with hydrogenated zigzag edges with respect to their size and edge atom species. The effective screening medium (ESM) method was adopted to investigate the electronic structure of triangular h-BN nanoflakes under an external electric field^50^. To inject holes into the triangular flakes with hydrogenated N zigzag edges, we considered a field-effect-transistor structure, in which a planar counter metal electrode described by the ESM having an infinite relative permittivity was separated by a 0.35 nm vacuum spacing from the nanoflakes. In contrast, an open boundary condition was imposed at the opposite cell boundary described by a relative permittivity of 1 with a vacuum spacing of 0.35 nm from the center the flakes. During the electronic structure calculations under an electric field, the geometric structures of the triangular h-BN nanoflakes were fixed to their optimized structure without an electric field.
